# The Association between Free Sugars Consumption and Laryngopharyngeal Reflux: A Cross-Sectional Study among Chinese Adolescents

**DOI:** 10.3390/nu13093012

**Published:** 2021-08-28

**Authors:** Fang Li, Qian Lin, Qiping Yang, Yue Xi, Hanmei Liu, Jing Luo, Yufeng Ouyang, Minghui Sun, Cuiting Yong, Caihong Xiang, Jing Deng

**Affiliations:** 1 Department of Epidemiology and Health Statistics, Xiangya School of Public Health, Central South University, Changsha 410078, China; lifang_csu@csu.edu.cn; 2 Hunan Provincial Key Laboratory of Clinical Epidemiology, Changsha 410078, China; 3 Department of Nutrition Science and Food Hygiene, Xiangya School of Public Health, Central South University, Changsha 410078, China; linqian@csu.edu.cn (Q.L.); yangqiping12@csu.edu.cn (Q.Y.); xiyue0404@csu.edu.cn (Y.X.); hanmeiliu@csu.edu.cn (H.L.); luojing2546@csu.edu.cn (J.L.); oyyf0102@csu.edu.cn (Y.O.); sun.1234@csu.edu.cn (M.S.); yongcuiting@csu.edu.cn (C.Y.); xch0622@csu.edu.cn (C.X.)

**Keywords:** laryngopharyngeal reflux, free sugars, Chinese adolescents

## Abstract

There is a lack of evidence to show prevalence of laryngopharyngeal reflux (LPR) and the association between LPR and dietary factors. Adolescents consume the most amount of free sugars among the Chinese population. We conducted this study to investigate the prevalence of LPR in Chinese adolescents and explore the association between free sugars consumption and LPR. A cross-sectional study was conducted on 1517 middle school students in Hunan, China. An online questionnaire was applied to collect data on the condition of LPR, consumption of free sugars and other self-reported covariates. Height, weight and waist circumference were collected by anthropometric measurements. Logistic regression was applied to assess the association between LPR and free sugars consumption. The mean and standard deviation of free sugars consumption was 53.14 ± 44.75 (g/d). The prevalence of LPR was 8.11%. A positive association was observed between LPR and higher free sugars consumption after adjusted multiple covariates, with adjusted odds ratio (95% confident interval) of 1.656 (1.125–2.438). The prevalence of LPR among Chinese adolescents was high. Further analytic studies with strict design are required to test the association between LPR and free sugar consumption. Systematic strategies and policies should to be developed to reduce the intake of free sugars in order to prevent LPR.

## 1. Introduction

Laryngopharyngeal reflux (LPR) is an inflammatory condition of the upper aerodigestive tract tissues related to the direct and indirect effects of gastroduodenal content reflux, which may induce morphologic changes in the upper aerodigestive tract [1,2]. Evidence even shows that LPR oriented chronic laryngeal irritation may explain the development of laryngeal carcinoma [3]. The symptoms of LPR vary and include minor malaise, such as dysphonia or hoarseness, cough, throat clearing, dysphagia and severe conditions such as edema, granuloma, erythema, and pseudosulcus of laryngeal [4]. The symptoms related to LPR prevail among 10 to 30% of otolaryngology patients, making up half of laryngology practices in Western Countries [1,4]. Spantideas and colleagues reported the prevalence of LPR to be 18.8% in the Greek general population [5]. A study conducted in 2016 reported the prevalence of LPR ranged from 2.85% to 6.29% among different age groups in a city located in the Eastern China [6]. Our previous study showed that the prevalence of LPR was 8.1% of Chinese college students [7]. Evidence shows that the LPR can be alleviated by lifestyle intervention [8], thus it is important to detect the risk factors to prevent LPR.

Free sugars include monosaccharides and disaccharides added to foods and beverages by the manufacturer, cook or consumer, and sugars naturally present in honey, syrups, fruit juices and fruit juice concentrates [9]. According to the Chinese Nutrition and Health Survey, the consumption of free sugars has been rising, reaching 18.8 g/d in 2012 [10]. The highest consumption was found in the 12 to 17 year age group (22.5 g/d), which indicated the urgency to control free sugar intake among adolescents [10]. The excessive consumption of free sugars contributes to unhealthy diet, weight gain, various noncommunicable diseases and dental problems [11]. Previous studies have investigated the association between diet and gastroesophageal reflux disease (GERD), a disease that shared similar symptoms and pathophysiologic characteristics with LPR, as well as Barrett’s esophagus (BE), a complication of GERD [12,13]. Evidence shows that limiting dietary intake of free sugars may reduce the risk of developing GERD and BE [12,13]. Yet, only a few epidemiological studies have been published relating to the association between LPR and dietary factors. A study assessed the impact of diet on the occurrence of proximal reflux episodes in 85 patients with LPR and concluded that the consumption of high-fat, low-protein, high-sugar, acid foods, and beverages was associated with a higher number of proximal reflux episodes [14]. However, in the general population, research focusing on this issue is rare.

Faced with high consumption of free sugars among Chinese adolescents and the lack of evidence on the association between LPR and free sugars intake, we conducted this study in order to investigate the prevalence of LPR in Chinese adolescents and explore the association between free sugars consumption and LPR.

## 2. Materials and Methods

### 2.1. Ethical Approval

The study was approved by the ethics review committee of the Xiangya School of Public Health, Central South University (XYGW-2019-025) and conducted according to the guidelines of the Declaration of Helsinki. Written informed consent was obtained from parents or caregivers involved in the study before the survey.

### 2.2. Study Design and Participants

From March to July 2019, a cross-sectional study was conducted in ten middle schools, which were selected by a two-stage random cluster sampling process in five districts of Changsha City, Hunan Province. For stage one, two middle schools were sampled from each district; for stage two, two classes were sampled from the 7th and 8th Grade, respectively, from each sampled middle school in stage one. All students from each sampled class were involved in this study after written consent was provided by their parents or caregivers.

The inclusion criteria were as follows: (1) schools had no less than 500 current students; and (2) parents or caregivers of students gave written informed consent. The exclusion criterion was the students who were unable to read or write in order to finish the questionnaire.

Calculated by PASS software (version 11.0 for Windows; NCSS LLC, Kaysville, UT, USA), the required sample size was 1338, with a prevalence of LPR among Chinese adolescent of 8.1% and an allowable error of 3%. We recruited 1628 students in this study, and 1517 students (93.2%) who finished the questionnaires were the final valid samples.

### 2.3. Data Collection

The outcome of interest was the condition of LPR. A self-developed online questionnaire was applied to collect information on the condition of LPR, consumption of free sugars and other self-reported covariates. Height, weight and waist circumference (WC) were collected by anthropometric measurements. A pilot study was conducted among 150 middle school students to test the feasibility of the questionnaire.

#### 2.3.1. Condition of Laryngopharyngeal Reflux

The condition of LPR was assessed by the reflux symptom index (RSI) [15]. RSI is a self-reported tool with 9 items concerning LPR symptoms, including (1) hoarseness or voice problem, (2) throat clearing, (3) excess throat mucus or postnasal drip, (4) difficulty swallowing, (5) coughing after eating or lying down, (6) breathing difficulties or choking spells, (7) troublesome or annoying cough, (8) sensation of something sticking or a lump in the throat, and (9) heartburn, chest pain, indigestion, or stomach acid coming up. Each item of symptoms is graded by a 0- to 5-point scale from asymptomatic to very severe. The sum of each item ranges from 0 to 45 points, and RSI greater than 13 is considered abnormal and likely to be LPR positive. The RSI is shown in Table A1.

#### 2.3.2. Consumption of Free Sugars

Consumption of free sugars was assessed by a food frequency questionnaire (FFQ) on sweetened drinks and foods. This FFQ was developed according to two previous studies conducted by the Center for Disease Control and Prevention of China [10,16]. The food items included in FFQ were adjusted according to the result of the pilot study. The frequencies and average amount of consumption of 11 types of drink and food were assessed, including sweetened drinks (carbonated drinks, bubble tea, tea drinks, juice or juice drinks, vegetable protein drinks, sports drinks), sweetened foods (biscuits/cakes, chocolates/candies, preserved fruits), honey and flavored milk/yogurt. Consumption frequencies of each drink and food items were reported and further transformed by following rules: 0 times/day = “once a month or less”, 0.14 times/day = “once a week”, 0.36 times/day = “2–3 times a week”, 0.64 times/day = “4–5 times a week” and 2 times/day = “2 times/day or more”. The average contents per serving (100 g or 100 mL) of free sugars in the mentioned above 11 types of drink and food (243 items in total) were collected via China CDC database [16]. The summary of the average contents of free sugars in each of the 11 types of drink and foods is shown in Table A2.

The daily intake of free sugars of a certain drink or food item was calculated by multiplying the frequencies and average amount of consumption of drink or food items. By adding up the daily free sugar intake of each drink or food item, the daily intake of free sugars of an individual was estimated. Formula 1 describes this estimation:

(1) $$Z=\overset{n}{\underset{i=1}{\sum }}f_{i}m_{i}C_{i}$$

where Z (g/d) was daily consumption of free sugars of an individual; $f_{i}$ (times/day) was the transformed frequency of consumption and $m_{i}$ (g or mL) was the average amount of each consumption of certain drink or food item; i (i = 1,2, ⋯, n). $C_{i}$ (g/100 g or g/100 mL) was the content of free sugars of drink or food item i (Table A2). If the free sugars content of some certain food or drink was not available from the database, the average content of this type of food or drink was used as the proxy. Then the students were further divided into two groups according to their daily consumption of free sugars: low-sugar group (<50 g/d) and high-sugar group (≥50 g/d) by the recommendation of WHO [11].

#### 2.3.3. Covariates

Demographic characteristics of students and their families were also collected by online questionnaire, including information on students (e.g., gender, age, ethnicity, number of siblings, grade, location, boarding or day students, left-behind student or not) and information on their family (parental education level, family income level).

We also estimated self-esteem using the Rosenberg self-esteem scale (SES) [17]. This is a tool with 10 items graded on a 4-point scale ranging from strongly agree to strongly disagree (Table A3). The value of items 1, 2, 4, 6 and 7 were: strongly agree = 4, agree = 3, disagree = 2, and strongly disagree = 1; while for items 3, 5, 8, 9 and 10 are reversed in valence. The score ranges from 10 to 40 and higher scores imply higher self-esteem. The upper and lower quartiles were applied to divide the participants into three groups: low self-esteem group (<P_25_), middle self-esteem group (P_25_–P_75_), and high self-esteem group (>P_75_).

Physical activity was measured using the International Physical Activity Questionnaire short form (IPAQ-SF) [18]. The IPAQ-SF comprises 4 generic items, collecting information on the time spent on vigorous activities, moderate activities, walking, and sitting over a week. The metabolic equivalent task (MET) level of participants was calculated and categorized into three levels: low, middle and high.

#### 2.3.4. Anthropometric Measurements

Height, weight and WC were measured by trained researchers. Calibrated standard height meters were applied to measure the height at head level with the participant standing barefoot and documented to the nearest 0.1 cm. Body composition analyzers (TANITA human body composition analyzer, BC-W02C) were applied to assess the weight and documented to the nearest 0.1 kg. WC was measured by a calibrated flexible non-stretch tape laid at the level of belly bottom and documented to the nearest 0.1 cm. The body mass index (BMI) of participants was calculated by weight and height, and further classified into normal, overweight or obese according to age- and gender- specified cut-offs of Chinese children and adolescents [19]. The WC was graded into normal (<P_75_), normal-high (P_75_–P_90_) and high (>P_90_) according to the age- and gender- specified cut-offs [20].

### 2.4. Statistical Analysis

For statistical description, if continuous variables were normally distributed, they were presented as mean and standard deviation; if not, they were presented using medians and interquartile ranges. Categorical variables were descripted by numbers and proportions. Continuous variables were compared using one-way ANOVA or Kruskal–Wallis tests and categorical variables were analyzed using Chi-square tests or Fisher’s exact tests, respectively.

The analyses of associations between LPR and consumption of free sugars were conducted by the Logistic regression and the association was descripted by odds ratios (OR) and correspondence 95% confidence intervals (CI). The receiver operating characteristic curve (ROC) and the area under the curve (AUC) were applied to assess the feasibility of the cut-off of 50 g/d free sugar consumption.

Missing values of categorical variables were processed by the multiple imputation method [21]. The Chi-square test was conducted among individuals without missing values to assess the effect of the missing values.

Statistical analyses were conducted by IBM SPSS (version 25.0). The significant level was *p* < 0.05 except where specifically mentioned.

## 3. Results

### 3.1. Characteristics and Free Sugars Consumption of Participants

Among 1517 participants, 53.3% (808 cases) of them were male. The age ranged from 12 to 14 years with mean and standard deviation of 13 ± 1 (years). The mean and standard deviation of free sugars consumption was 53.14 ± 44.75 (g/d), 43.2% of participants consumed more than 50 g of free sugars every day. The distribution of free sugars consumption was different among participants depending on grade, or whether they were boarding or day students (all *p* < 0.05). The prevalence of higher free sugars consumption among different characteristics is shown in Table 1.

### 3.2. Characteristics and Laryngopharyngeal Reflux Condition of Participants

The prevalence of LPR was 8.11% (123/1517), with 95% CI of 6.84–9.59%. The prevalence of LPR was different among participants with different ages, grades, or whether they were left-behind children or not, WC, and free sugars consumption (all *p* < 0.05). The details are shown in Table 2. The scores of each RSI items are shown in Table A4. Appendix A Table A6 presents the prevalence of LPR among different SES items.

We further conducted sensitivity analysis using the Chi-square test to estimate the impact of missing values (Table A5). The sensitivity analysis showed that the association between characteristics and RSI of participants after imputation were similar before imputation.

### 3.3. Association between Laryngopharyngeal Reflux Condition and Free Sugars Consumption

The LPR positive was regressed onto levels of free sugars consumption and other covariates with significantly different distribution in Table 2and Table A6. Different logistic regression models are established and shown in Table 3.

In Model 1, adolescents who consumed more than 50 g/d of free sugars were more likely to be reported as LPR positive compared with adolescents who consumed less than 50 g/d of free sugars, with adjusted OR (95% CI) of 1.757 (1.211–2.547). This association remained after adjustment for multiple covariates.

In Model 4, the adjusted OR (95% CI) was 1.656 (1.125–2.438) for participants who consumed 50 g/d more free sugars compared to participants who consumed less than 50 g/d of free sugars. We also found that participants who were older, who had a high WC level and who reported low self-esteem in some items of SES were more likely to report as LPR positive after adjustment for multiple covariates (Table 3, Model 4). Other detailed figures are presented in Table 3.

**Figure 1:**
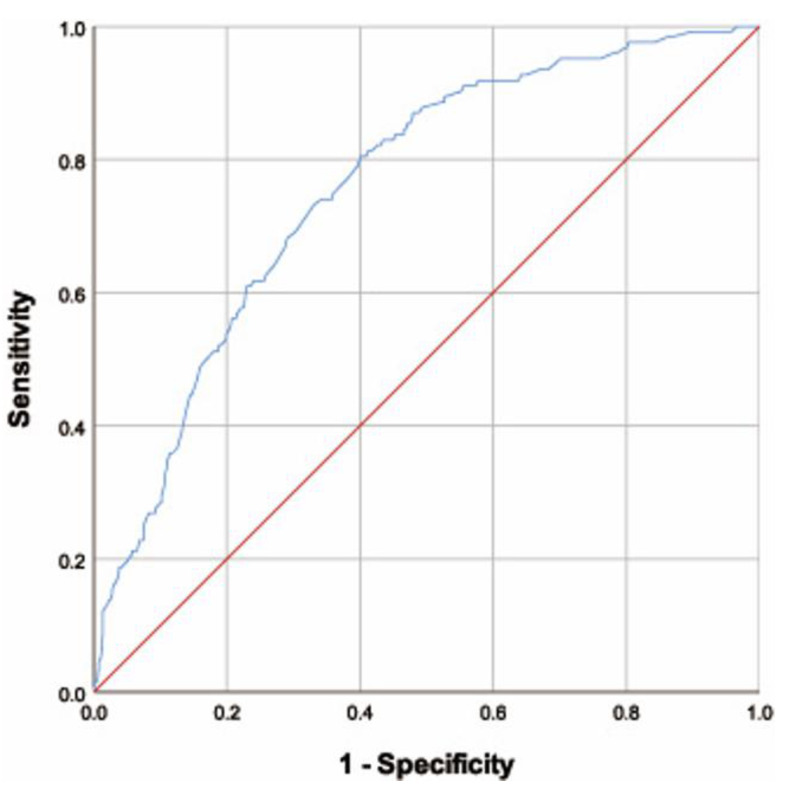
The receiver operating characteristic curve of the free sugars consumption and other covariates based on Model 4 of Table 3.

Furthermore, we conducted the ROC analysis based on the above mentioned Model 4 (Figure 1). The AUC was 0.758 (95% CI: 0.717–0.799), which indicated that the prediction model based on the cut-off value of 50 g/d of free sugars consumption was acceptable.

## 4. Discussion

In this study, we found that the prevalence of LPR was 8.11% among Chinese adolescents and was positively associated with free sugars consumption. The finding of this study may shed light on the management of LPR in adolescents.

The prevalence of LPR symptoms in this study was similar to a study conducted in 2019 among college students who ranged from 17 to 25 years old in Hunan Province (8.1%) [7]. The result of this study was higher than a study conducted in the Fuzhou City in China, which reported the age-specific prevalence of 2.85% to 6.29% in the general population and 2.85% among participants of 10 to 19 years old in 2015 [6]. Globally, the prevalence of LPR ranged widely in different countries. According to reports from a worldwide survey on otolaryngologists, it was estimated to range from 5% to 90% (mean: 23.7%) in the general population [22]. The vast discrepancy may partly be due to the atypia of symptoms and the lack of a universal standard of diagnostic for LPR. A systematic review conducted in 2018 summarized the studies that used the RSI to estimate LPR prevalence and observed heterogeneity due to various populations with different thresholds [1]. For example, an 18.8% prevalence of LPR was reported in Greek using the threshold of RSI ≥ 13, while a 34.4% prevalence of LPR was reported in England using the threshold of RSI > 10 [5,23]. To obtain the precise prevalence of LPR, the consensus of LPR diagnoses needs to be reached and a golden standard approach needs to be established to confirm LPR [24]. Further studies on the prevalence of LPR need to combine the subjective symptom assessment instruments with objective diagnostic tools, such as 24-h multichannel intraluminal impedance-pH metry (MII-pH metry), GI endoscopy, and methods for detection of pepsin in saliva [1,2,8].

Previous studies have shown that LPR was related to various dietary factors, such as alcohol, caffeine, tea, high-osmolality beverages, and fatty foods [25]. For example, Lechien and colleagues showed that the consumption of high-fat, low-protein, high-sugar, acid foods and beverages would exacerbate the symptoms of LPR patients [14]. In addition, Zalvan and colleagues reported that the percent reduction in RSI was significantly greater with dietary approaches (of alkaline water, a plant-based, Mediterranean-style diet, and standard reflux precautions) than with proton pump inhibitors (PPIs) [26]. They also suggested that the effect of dietary approaches on the RSI based on the proportion reaching a 6-point reduction in RSI is similar to treatment of PPIs [26].

For the first time, our study quantified free sugars consumption among Chinese adolescents and observed a positive association between the free sugars consumption and LPR risk. Moreover, the result indicated that the prediction model based on the cut-off value of 50 g/d of free sugars consumption was acceptable. Although there is no comparable direct evidence concerning the association between quantified consumption of free sugars and risk of LPR, the indirect evidence may shed light on this issue. A previous study showed a positive association between specific types of carbohydrates and GERD [12]. Furthermore, the disaccharides and starches were observed to be linked with increasing GERD symptoms [27,28,29,30]. This may be due to induced neurohormonal release and lower esophageal sphincter relaxation, and the fermentation process during carbohydrate digestion [31]. In other words, the consumption of free sugars (mainly monosaccharides and disaccharides) may exacerbate the reflux of stomach contents. LPR shared similar pathogenesis with GERD; the damage on the upper aero-digestive tract mucosa of LPR patients mainly due to the reflux of pepsin, bile salts and other gastrointestinal proteins [1]. Thus, we assumed that the association between free sugars consumption and risk of LPR may share the same mechanism of GERD.

We also observed a significant positive association between age, WC and LPR (Table 3, Model 4). These findings were in line with previous studies. We observed an increased risk of LPR among adolescents of senior age. According to the research conducted in Fuzhou, the prevalence of LPR firstly increased from 10 to 19 year olds, reaching a peak of 6.29% in 30 to 39 year olds among the Chinese general population [6]. The WC reflected obesity, which was also a risk factor to LPR [32,33]. Lechien and colleagues observed 262 patients with LPR-related symptoms and positive LPR diagnosis at the hypopharyngeal–esophageal MII-pH [34]. They found that obese patients reported a significantly higher prevalence of GERD, acid LPR, and a more severe LPR disease regarding the number of pharyngeal reflux events, reflux symptom score (RSS) and reflux sign assessment (RSA) [34].

The associations between some items of the Rosenberg self-esteem scale and LPR were observed: a lower score of two self-esteem items was associated with higher risk of LPR (Table 3, Model 4). We hypothesized that this was related to personal psychological characteristics: low self-esteem was associated with a high level of anxiety, stress and depression [35]. The association between psychological characteristics and LPR is controversial with only a few researchers discussing this issue [7,36,37]. In our previous study, we observed depressive symptoms to be positively associated with LPR symptoms [7]. Furthermore, Li and colleagues found that scores of anxiety and depressive symptoms in the LPR group were statistically significant and higher than those in the non-LPR group [36]. However, Mesallam and colleagues did not observe association between psychological disorders and LPR and reported that psychological background of the LPR patients had no influence on patients’ self-perception of their reflux-related problems [37]. Yet, considering that stress and anxiety may lead to the autonomic nerve dysfunction and transient esophageal sphincter relaxation, the stress and anxiety management were recommended in practical treatment of LPR by primary care physicians [8].

The finding of this study is particularly important when considering the increase in risk factors for LPR in China, such as the increasing of free sugars consumption and prevalence of obesity [10,38,39,40,41]. In our previous study, we found out that the daily consumption of free sugars was 53.1 g among adolescents, and 43.2% of the participants consumed more than 50 g/d of free sugars [42]. A meta-analysis summarized the trends in overweight and obesity among children and adolescents in China from 1991 to 2015 and found that the prevalence of overweight and obesity increased from 5.0% and 1.7% in 1991–1995 to 11.7% and 6.8% in 2011–2015, respectively [41]. Thus, it is important to develop policies and strategies to reduce free sugars consumption, such as increasing public concern relating to adverse effects of free sugars, paying tax on sugar-sweetened beverages, regulating advertising and improving labeling [43,44,45], to lower the prevalence of overweight/obesity and prevent LPR. In addition, the model consisting of free sugars consumption and other covariates helped to shed light on the etiology of LPR. We detected various potential risk factors of LPR in this study; controlling these risk factors, shows promise in lowering the prevalence of LPR among Chinese adolescents.

This study has some merits. With a sufficient sample size, for the first time we investigated the prevalence of LPR among Chinese adolescents and assessed the association between free sugars consumption and the risk of LPR, along with other covariates. In addition, strict quality control process was adopted. Weight, height and WC were measured by uniformly trained researchers, which improved the reliability of the anthropometric data. Pictures of sugar sweetened foods and beverages were shown to participants to improve the accuracy of recalling consumption. However, there are several limitations. Firstly, for LPR diagnosis, we only applied the subjective symptom assessment instruments without objective diagnostic tools, which may lead to the possibility of misclassification. However, under the circumference that no golden standard diagnosis approach has been established to confirm LPR, RSI was a feasible choice for epidemiologic studies with the relative large sample size. The reliability and validity of RSI is guaranteed and is easy to administer [15]. Secondly, although we tried our best to control confounders and adjusted various covariates in our analysis, we failed to evaluate some other dietary and lifestyle habits, which may be potential risk factors for LPR, such as consumption of coffee and tea, emotional eating and dietary patterns [1,7,46]. Thirdly, some missing values may bias the results. Nonetheless, the sensitivity analysis showed that the association between characteristics and RSI of participants after imputation were similar before imputation. Fourthly, it was disappointing that we failed to discuss all the foods that contained free sugars due to the limited food items in FFQ. However, the food items included in the FFQ of this study consisted of the main source of free sugars consumption of the target population [10]. Furthermore, the food items in the FFQ were adjusted according to the result of the pilot study with the target population. Thus, we were confident that the result of this FFQ represented the condition of free sugars consumption of middle school students. While numbers of foods containing free sugars was growing, tools specializing in free sugars consumption assessment are required.

## 5. Conclusions

In this study, we found that the prevalence of LPR was 8.1% among adolescents in Hunan, which was relatively high when compared with the peers in China. A positive association was observed between free sugars consumption and risk of LPR. Further analytic studies with strict design are required to test this association. Systematic strategies and policies should to be developed to reduce the intake of free sugars in order to prevent LPR.

## Figures and Tables

**Table 1:** Characteristics and free sugars consumption of participants (*n* = 1517).

Variables		<50 g (*n* = 861)		≥50 g (*n* = 656)		*p* Value
		Case	%	Case	%	
Gender	Male	444	55.0	364	45.0	0.130
	Female	417	58.8	292	41.2	
Age (year)	12	242	61.1	154	38.9	0.085
	13	403	56.2	314	43.8	
	14	216	53.5	188	46.5	
Grade	Grade 1	469	59.8	315	40.2	0.013
	Grade 2	392	53.5	341	46.5	
Ethnicity	Han (Majority)	818	56.5	630	43.5	0.340
	Minority	43	62.3	26	37.7	
Location	Urban	571	56.3	443	43.7	0.619
	Rural	290	57.7	213	42.3	
Single child	No	468	55.8	371	44.2	0.393
	Yes	393	58.0	285	42.0	
Boarding or day students	Day students	807	57.7	592	42.3	0.012
	Boarding students	54	45.8	64	54.2	
Left-behind child	Yes	36	62.1	22	37.9	0.405
	No	825	56.5	634	43.5	
Number of siblings	0	396	60.0	264	40.0	0.065
	1	312	53.2	274	46.8	
	2	121	58.5	86	41.5	
	3 and more	32	50.0	32	50.0	
Parental education level	Primary school and below	44	58.7	31	41.3	0.822
	Primary middle school	255	55.3	206	44.7	
	Senior middle school	317	58.1	229	41.9	
	College and above	245	56.3	190	43.7	
Family income level	Low	364	59.1	252	40.9	0.168
	Middle	311	56.6	238	43.4	
	High	186	52.8	166	47.2	
Weight status	Normal weight	616	55.8	487	44.2	0.492
	Overweight	142	58.7	100	41.3	
	Obesity	103	59.9	69	40.1	
Waist circumference	Normal	446	54.3	376	45.7	0.100
	Normal-high	195	59.3	134	40.7	
	High	220	60.1	146	39.9	
Activity level	Low	253	56.9	192	43.1	0.176
	Middle	427	58.7	301	41.3	
	High	181	52.6	163	47.4	
Self-esteem level	Low	145	53.1	128	46.9	0.278
	Middle	442	58.5	313	41.5	
	High	274	56.0	215	44.0	

**Table 2:** Characteristics and laryngopharyngeal reflux condition of participants (*n* = 1517).

Variables		LPR Negative (*n* = 1394)		LPR Positive (*n* = 123)		*p* Value
		Case	%	Case	%	
Gender	Male	751	92.9	57	7.1	0.108
	Female	643	90.7	66	9.3	
Age(year)	12	382	96.5	14	3.5	<0.001
	13	644	89.8	73	10.2	
	14	368	91.1	36	8.9	
Grade	Grade 1	738	94.1	46	5.9	0.001
	Grade 2	656	89.5	77	10.5	
Ethnicity	Han (Majority)	1329	91.8	119	8.2	0.472
	Minority	65	94.2	4	5.8	
Location	Urban	935	92.2	79	7.8	0.520
	Rural	459	91.3	44	8.7	
Single child	No	767	91.4	72	8.6	0.452
	Yes	627	92.5	51	7.5	
Boarding or day students	Day students	1284	91.8	115	8.2	0.582
	Boarding students	110	93.2	8	6.8	
Left-behind child	Yes	49	84.5	9	15.5	0.047 *
	No	1345	92.2	114	7.8	
Number of siblings	0	615	93.2	45	6.8	0.333
	1	532	90.8	54	9.2	
	2	187	90.3	20	9.7	
	3 and more	60	93.8	4	6.3	
Parental education level	Primary school and below	69	92.0	6	8.0	0.909
	Primary middle school	421	91.3	40	8.7	
	Senior middle school	501	91.8	45	8.2	
	College and above	403	92.6	32	7.4	
Family income level	Low	572	92.9	44	7.1	0.453
	Middle	503	91.6	46	8.4	
	High	319	90.6	33	9.4	
Weight status	Normal weight	1019	92.4	84	7.6	0.261
	Overweight	216	89.3	26	10.7	
	Obesity	159	92.4	13	7.6	
Waist circumference	Normal	772	93.9	50	6.1	0.007
	Normal-high	295	89.7	34	10.3	
	High	327	89.3	39	10.7	
Activity level	Low	399	89.7	46	10.3	0.083
	Middle	672	92.3	56	7.7	
	High	323	93.9	21	6.1	
Self-esteem level	Low	249	91.2	24	8.8	0.206
	Middle	703	93.1	52	6.9	
	High	442	90.4	47	9.6	
Consumption of free sugars	<50 g/d	807	93.7	54	6.3	0.003
	≥50 g/d	587	89.5	69	10.5	

* Tested by the Fisher’s exact test.

**Table 3:** Association between laryngopharyngeal reflux condition and free sugars consumption (*n* = 1517).

Models	Variable	OR	95% CI	*p* Value
Model 1 ^a^	Consumption of free sugars < 50 g/d	Ref.		
	Consumption of free sugars ≥ 50 g/d	1.757	1.211–2.547	0.003
Model 2 ^b^	Consumption of free sugars < 50 g/d	Ref.		
	Consumption of free sugars ≥ 50 g/d	1.793	1.230–2.615	0.002
	12 years old	Ref.		
	13 years old	3.027	1.680–5.454	<0.001
	14 years old	2.652	1.400–5.023	0.003
	Left-behind child	Ref.		
	Not left-behind child	0.458	0.215–0.976	0.043
	Normal waist circumference	Ref.		
	Normal-high waist circumference	1.845	1.160–2.936	0.010
	High waist circumference	1.907	1.224–2.972	0.004
Model 3 ^c^	Consumption of free sugars < 50 g/d	Ref.		
	Consumption of free sugars ≥ 50 g/d	1.794	1.229–2.618	0.002
	12 years old	Ref.		
	13 years old	3.014	1.672–5.434	<0.001
	14 years old	2.631	1.388–4.995	0.003
	Left-behind child	Ref.		
	Not left-behind child	0.463	0.217–0.992	0.048
	Normal waist circumference	Ref.		
	Normal-high waist circumference	1.848	1.160–2.943	0.010
	High waist circumference	1.907	1.223–2.974	0.004
	Low activity level	Ref.		
	Middle or high activity level	0.673	0.456–0.994	0.047
Model 4 ^d^	Consumption of free sugars < 50g/d	Ref.		
	Consumption of free sugars ≥ 50g/d	1.656	1.125–2.438	0.011
	12 years old	Ref.		
	13 years old	2.830	1.555–5.149	0.001
	14 years old	2.670	1.395–5.110	0.003
	Normal waist circumference	Ref.		
	Normal-high waist circumference	1.747	1.086–2.810	0.022
	High waist circumference	1.758	1.113–2.779	0.016
	I am able to do things as well as most other people—strongly disagree	Ref.		
	I am able to do things as well as most other people—disagree	3.502	1.587–7.730	0.002
	I am able to do things as well as most other people—agree	2.167	1.075–4.366	0.031
	I am able to do things as well as most other people—strongly agree	1.493	0.783–2.848	0.223
	All in all, I am inclined to think that I am a failure—strongly disagree	Ref.		
	All in all, I am inclined to think that I am a failure—disagree	2.665	1.373–5.174	0.004
	All in all, I am inclined to think that I am a failure—agree	4.381	2.266–8.468	<0.001
	All in all, I am inclined to think that I am a failure—strongly agree	7.031	3.433–14.397	<0.001

^a^ Crude OR; ^b^ adjusted for age, left-behind child or not, waist circumference; ^c^ adjusted for age, left-behind child or not, waist circumference, self-esteem level, activity level; ^d^ adjusted for age, left-behind child or not, waist circumference, items of the Rosenberg self-esteem scale presented in Table A6, activity level, variables are selected by stepwise forward (LR, *α*_in_ = 0.05, *α*_out_ = 0.10).

## Data Availability

The data that support the findings of this study are not publicly available due to the data containing information that could compromise participant privacy but are available from the corresponding author on reasonable request.

## Appendix A

**Table A1:** The Reflux Symptom Index (RSI).

Within the Last Month, How Did the Following Problems Affect You? Circle the Appropriate Response.	0 = No Problem 5 = Severe Problem					
1. Hoarseness or a problem with your voice	0	1	2	3	4	5
2. Clearing your throat	0	1	2	3	4	5
3. Excess throat mucus or postnasal drip	0	1	2	3	4	5
4. Difficulty swallowing food, liquids, or pills	0	1	2	3	4	5
5. Coughing after you ate or after lying down	0	1	2	3	4	5
6. Breathing difficulties or choking episodes	0	1	2	3	4	5
7. Troublesome or annoying cough	0	1	2	3	4	5
8. Sensations of something sticking in your throat or a lump in your throat	0	1	2	3	4	5
9. Heartburn, chest pain, indigestion, or stomach acid coming up	0	1	2	3	4	5
**TOTAL**						

**Table A2:** The average free sugars content of drink and food item.

Drink and Food Types	Quantity	Items	Average Free Sugars (g)
Sodas	100 mL	9	9.79
Bubble tea	100 mL	7	7.89
Tea drinks	100 mL	6	4.31
Fruit or natural juice	100 mL	21	10.47
Vegetable protein beverage	100 mL	19	7.14
Sports drink	100 mL	6	8.22
Flavored milk/yogurt	100 mL	29	12.28
Biscuits, cakes	100 g	118	17.50
Chocolate, confectionery	100 g	14	44.63
Fruit preserves	100 g	12	55.04
Honey	100 g	2	73.00

**Table A3:** The Rosenberg Self-Esteem Scale.

Instruction: Please Record the Appropriate Answer for Each Item, Depending on whether You Strongly Agree, Agree, Disagree, or Strongly Disagree with It.	
I feel that I am a person of worth, at least on an equal basis with others.	1 = Strongly disagree 2 = Disagree 3 = Agree 4 = Strongly agree
I feel that I have a number of good qualities.	1 = Strongly disagree 2 = Disagree 3 = Agree 4 = Strongly agree
All in all, I am inclined to feel that I am a failure.	1 = Strongly disagree 2 = Disagree 3 = Agree 4 = Strongly agree
I am able to do things as well as most other people.	1 = Strongly disagree 2 = Disagree 3 = Agree 4 = Strongly agree
I feel I do not have much to be proud of.	1 = Strongly disagree 2 = Disagree 3 = Agree 4 = Strongly agree
I take a positive attitude toward myself.	1 = Strongly disagree 2 = Disagree 3 = Agree 4 = Strongly agree
On the whole, I am satisfied with myself.	1 = Strongly disagree 2 = Disagree 3 = Agree 4 = Strongly agree
I wish I could have more respect for myself.	1 = Strongly disagree 2 = Disagree 3 = Agree 4 = Strongly agree
I certainly feel useless at times.	1 = Strongly disagree 2 = Disagree 3 = Agree 4 = Strongly agree
At times I think I am no good at all.	1 = Strongly disagree 2 = Disagree 3 = Agree 4 = Strongly agree

**Table A4:** The scores of each Reflux Symptom Index items.

Items	0 Point		1 Point		2 Points		3 Points		4 Points		5 Points	
	Case	%	Case	%	Case	%	Case	%	Case	%	Case	%
Hoarseness or a problem with your voice	1080	71.2	240	15.8	109	7.2	45	3.0	20	1.3	23	1.5
Clearing your throat	1035	68.2	250	16.5	126	8.3	62	4.1	19	1.3	25	1.6
Excess throat mucus or postnasal drip	944	62.2	257	16.9	144	9.5	85	5.6	42	2.8	45	3.0
Difficulty swallowing food, liquids, or pills	1310	86.4	125	8.2	43	2.8	14	0.9	10	0.7	15	1.0
Coughing after you ate or after lying down	1230	81.1	170	11.2	59	3.9	37	2.4	10	0.7	11	0.7
Breathing difficulties or choking episodes	1324	87.3	97	6.4	48	3.2	24	1.6	12	0.8	12	0.8
Troublesome or annoying cough	1094	72.1	230	15.2	95	6.3	53	3.5	24	1.6	21	1.4
Sensations of something sticking in your throat or a lump in your throat	1126	74.2	208	13.7	91	6.0	43	2.8	22	1.5	27	1.8
Heartburn, chest pain, indigestion, or stomach acid coming up	1180	77.8	162	10.7	85	5.6	45	3.0	18	1.2	27	1.8

**Table A5:** Sensitivity analysis of the association between characteristics and RSI of participants.

Variables		Original				*p* Value	Imputed				*p* Value
		LPR Negative		LRP Positive			LPR Negative		LRP Positive		
		Case	%	Case	%		Case	%	Case	%	
Parental education level ^a^	Primary school and below	61	92.4	5	7.6	0.810	69	92.0	6	8.0	0.909
	Primary middle school	345	90.8	35	9.2		421	91.3	40	8.7	
	Senior middle school	418	91.1	41	8.9		501	91.8	45	8.2	
	College and above	348	92.6	28	7.4		403	92.6	32	7.4	
Family income level ^b^	Low	375	91.9	33	8.1	0.960	572	92.9	44	7.1	0.453
	Middle	338	91.4	32	8.6		503	91.6	46	8.4	
	High	227	91.5	21	8.5		319	90.6	33	9.4	

^a^ Parental education level missing of 236 cases; ^b^ Family income level missing of 491 cases.

**Table A6:** The laryngopharyngeal reflux condition and items of Rosenberg Self-Esteem Scale.

Variables		LPR Negative (*n* = 1394)		LPR Positive (*n* = 123)		*p* Value
		Case	%	Case	%	
I feel that I am a person of worth, at least on an equal basis with others	Strongly disagree	112	86.2	18	13.8	0.001
	Disagree	189	86.7	29	13.3	
	Agree	729	93.1	54	6.9	
	Strongly agree	364	94.3	22	5.7	
I feel that I have a number of good qualities	Strongly disagree	95	85.6	16	14.4	<0.001
	Disagree	233	87.6	33	12.4	
	Agree	740	92.7	58	7.3	
	Strongly agree	326	95.3	16	4.7	
All in all, I am inclined to feel that I am a failure	Strongly disagree	543	96.1	22	3.9	<0.001
	Disagree	554	92.5	45	7.5	
	Agree	204	84.0	39	16.0	
	Strongly agree	93	84.5	17	15.5	
I am able to do things as well as most other people	Strongly disagree	81	82.7	17	17.3	<0.001
	Disagree	207	86.3	33	13.8	
	Agree	765	92.8	59	7.2	
	Strongly agree	341	96.1	14	3.9	
I feel I do not have much to be proud of	Strongly disagree	263	94.9	14	5.1	0.015
	Disagree	462	93.5	32	6.5	
	Agree	531	90.0	59	10.0	
	Strongly agree	138	88.5	18	11.5	
I take a positive attitude toward myself	Strongly disagree	73	85.9	12	14.1	0.004
	Disagree	230	88.8	29	11.2	
	Agree	754	92.0	66	8.0	
	Strongly agree	337	95.5	16	4.5	
On the whole, I am satisfied with myself	Strongly disagree	87	87.9	12	12.1	0.001
	Disagree	292	88.0	40	12.0	
	Agree	687	92.3	57	7.7	
	Strongly agree	328	95.9	14	4.1	
I wish I could have more respect for myself	Strongly disagree	68	93.2	5	6.8	0.810
	Disagree	114	89.8	13	10.2	
	Agree	735	92.0	64	8.0	
	Strongly agree	477	92.1	41	7.9	
I certainly feel useless at times	Strongly disagree	515	97.4	14	2.6	<0.001
	Disagree	487	92.4	40	7.6	
	Agree	293	86.9	44	13.1	
	Strongly agree	99	79.8	25	20.2	
At times I think I am no good at all	Strongly disagree	616	96.1	25	3.9	<0.001
	Disagree	475	91.3	45	8.7	
	Agree	204	87.2	30	12.8	
	Strongly agree	99	81.1	23	18.9	
